# Evolving information complexity of coarsening materials microstructures

**DOI:** 10.1038/s41598-023-49759-x

**Published:** 2023-12-16

**Authors:** J. M. Rickman, K. Barmak, B. Y. Chen, Matthew Patrick

**Affiliations:** 1 Department of Physics, Lehigh University, Bethlehem, PA 18015 USA; 2 Department of Materials Science and Engineering, Lehigh University, Bethlehem, PA 18015 USA; 3 Department of Applied Physics and Applied Mathematics, Columbia University, New York, NY 10027 USA; 4 Department of Computer Science and Engineering, Lehigh University, Bethlehem, PA 18015 USA

**Keywords:** Materials science, Theory and computation, Computational methods

## Abstract

The temporal evolution of microstructural features in metals and ceramics has been the subject of intense investigation over many years because deviations from normal grain growth behavior are ubiquitous and strongly dictate observed mechanical and magnetic properties. To distinguish among different grain growth scenarios, we examine the time evolution of the information content of both synthetic and experimental coarsening microstructures as quantified by both a computable information density (CID) and a spectral entropy along with selected metrics and measures of shared information and interaction strength. In these approaches, microstructural evolution is described in terms of two time series representations, namely: (1) strings and their compressed counterparts that reflect the information contained in the configuration of a system over time, and (2) the spectra of graph Laplacians that embody the information contained in a coarsening grain network. These approaches permit one to characterize dynamically evolving microstructures and to identify correlation times associated with different coarsening scenarios. Moreover, as the information content of a system is a proxy for the entropy, a thermodynamic description of grain growth is also described.

## Introduction

Internal interfaces, such as grain boundaries, are prevalent in metals and ceramics and they often influence material properties that dictate structural, functional, and battery applications^1–4^. When such materials are held at sufficiently high temperatures, the grain structure evolves via coarsening with a concomitant decrease in the excess grain-boundary free energy^5^. Since this kinetic process governs the temporal evolution of key microstructural features, it has been the subject of intense investigation over many years^6,7^. In particular, one is often interested in identifying factors that result in extreme events characterized, for example, by very large, possibly non-equiaxed grains. Despite considerable effort, however, it is fair to say that the interplay among mechanisms that result in such events remains poorly understood and that there is little consensus as to which specific microstructural features define this behavior.

From these grain-growth studies, one finds that it is useful to benchmark coarsening behavior relative to normal grain growth (NGG). In the NGG regime the associated grain-size distribution obeys a simple scaling relation that follows from statistical self-similarity^5,8^. In other regimes this self-similar behavior breaks down, especially at late times. For example, one sometimes observes abnormal grain growth (AGG) in which a minority of grains having boundary energies and/or mobilities that differ substantially from the majority of grains becomes relatively large and overtakes the surrounding “normal” matrix^9^. This rapid growth characteristic of AGG may occur, in some situations, due to the presence of impurity excesses, such as Ca or Si in Al$_{2}$O$_3$^10^, or as a result of grain-boundary complexion transitions in which a first-order, phase-like transformation occurs that produces a new interfacial state^11–16^ with changes in boundary structure and/or chemistry. We note that AGG is ubiquitous and often deleterious as it leads to heterogeneous microstructures and an associated degradation in mechanical properties (e.g., strength hardness)^17^. In some cases, however, AGG is advantageous as, for example, elongated grains can enable crack-tip bridging with a resulting improvement in fracture toughness^18,19^. In short, an understanding of deviations from NGG and, in particular, the onset of AGG is crucial for predicting evolving materials microstructures and associated properties.

In this work, we examine the kinetics of evolving synthetic and experimental microstructures as quantified by their embodied information. More specifically, our focus here is on the time evolution of the information content of coarsening microstructures as quantified by selected metrics and measures of shared information and interaction strength. Two interrelated approaches will be employed, one based on the Kolmogorov complexity calculated with compressed data and the other based on the spectrum of a graph Laplacian that characterizes the microstructural network. These approaches permit one to identify, in a *dynamic* context, deviations from NGG and to obtain correlation times associated with different coarsening scenarios. We assert that a dynamic assessment of such deviations is superior to one based on anecdotal, static microstructural observations and provides a foundation for a thermodynamic description of coarsening^20^.

The field of information theory originated with a paper by Shannon^21^ on communication theory and has had an impact in such disparate disciplines as thermodynamics and computer science^22^. It asserts that the entropy describes the ultimate data compression and that the Kolmogorov complexity is a proxy and “conceptual precursor” for the entropy^22^. A simple example illustrates the intuitive connection between data compression and entropy. Consider a string of letters. If the letters are highly repetitive, the string can be described with a relatively short string that is repeated many times and the associated compressibility is relatively high. (If the characters of that string represent elements of a microstructure, then one could presume that the microstructure itself is highly repetitive, as would be the case if the average grain size is large.) Conversely, if it is not possible to find repetitions or other compression strategies that shorten the string substantially, then the string has a high degree of randomness and its associated entropy is high.

The Kolmogorov complexity, as represented by the so-called “computable information density”^23^, has recently been used to characterize the behavior of prototypical systems outside equilibrium. However, to our knowledge, the full machinery of information theory has not been applied to the study of grain growth and associated anomalies, non-equilibrium phenomena of considerable technological relevance. We will exploit Kolmogorov complexity, spectral network analysis and extreme-value statistics here to quantify the kinetics of grain growth and, in so doing, highlight the power and utility of these tools in this context and, in addition, make a connection with thermodynamic analyses of time-varying network complexity^24^.

## Results

### Kolmogorov complexity and spectral entropy for microstructures

Consider a discretized microstructure given on a $\ell ^{d}$ *d*-dimensional simple (hyper)cubic lattice of voxels. Each voxel is numbered from 1 to *Q* corresponding to one of the *Q* associated grains. (In this work we will consider microstructures in $d=2$ and 3 dimensions.) We examine both synthetic and metallic thin-film experimental microstructures here and, in the former case, employ a complexion nucleation mechanism to induce changes in coarsening behavior. For the evolving synthetic microstructures, two prototypical grain growth scenarios following from different complexion nucleation assumptions are modeled here using a discrete, coarse-grained microstructural (Potts) model, namely: (Scenario A) no complexion transitions, leading to isotropic, NGG that is statistically self-similar^8^ and (Scenario B) complexions that are spatially randomly nucleated on grain boundaries in addition to complexions that are propagated from a previously transitioned grain boundary^25^. In this latter case, complexion nucleation alters grain-boundary mobilities, resulting in deviations from NGG and, in some instances, AGG. We note that the density of seed points and, more generally, the nucleation conditions clearly dictate the onset for AGG. Further details regarding the simulations may be found in the Methods section and an analysis of the experimental microstructures may be found in the Discussion section below.

It is convenient to describe the information content in a given microstructure in terms of two quantities, namely: 1.) its Kolmogorov complexity^26,27^, the counterpart of the entropy in information theory, and 2.) its spectral (von Neumann) complexity^28^, the latter a function of the spectrum of a graph Laplacian that reflects the topology of the grain network. These complexities embody the connectivity of the microstructure, and therefore spatial correlations among grains, making them superior in this context to many other descriptors, such as the Shannon entropy^29^ based on a grain-size distribution. Our aim here is to employ these two quantities to describe the time evolution of information that attends coarsening under different grain growth scenarios and, in so doing, to distinguish between these scenarios. In the first case, the complexity is approximated in terms of an entropy proxy, known as the computable information density (*CID*), by writing a string, denoted by *x*, representing the microstructure to a file and then comparing the length of this file, $\vert x \vert$, to the length of its compressed counterpart, ${\mathcal {C}} \left( x \right)$.

Then, the density $S_{CID} \left( x \right) {:}{=}{\mathcal {C}} \left( x \right) /\vert x \vert$, the ratio of the lossless compressed to the uncompressed string lengths^23^. To construct the requisite one-dimensional string corresponding to a given microstructure that preserves to some degree intrinsic, nearest-neighbor microstructural information, it is convenient to employ a Hilbert scan of the lattice^30^. This scan produces a one-to-one mapping between *d*-dimensional data (i.e., the voxel values summarizing the microstructure) and a one-dimensional representation along a Hilbert curve^31^. More specifically, the Hilbert curve sinuously traverses the elements of a matrix of integers that represent a microstructural snapshot at some point in time. (Such curves may be conveniently constructed using a built-in function found, for example, in Mathematica^32^). The resulting one-dimensional string of integers is then stored in a file whose length corresponds to $\vert x \vert$. This file is then compressed utilizing a lossless compressor such as xz resulting in a new, typically smaller file with corresponding length ${\mathcal {C}} \left( x \right)$. (This compressor and its variants may be invoked as a standard Unix command. Additional details may be found in the “Methods” section.) For calculations of compression distances and interaction information, it is necessary to sometimes concatenate first uncompressed files as will be described below.

In the second case, the *Q* grains comprise the vertices of an undirected graph, each vertex thereby corresponding to the collection of voxels having the same spin value (i.e., belonging to the same grain). The graph edges connect a given grain to its nearest neighbors. The complexity is then a function of the spectrum of the associated, normalized graph Laplacian, *L*, and can be expressed as

1 $$\begin{aligned} S_{spec} = - \sum _{i=1}^{m} \lambda _{i} \log _{2}{\lambda _{i}}, \end{aligned}$$

where the $\{ \lambda _{i} \}$ $(i=1,2, \cdots , m)$ are the *m* eigenvalues of *L*. For the (connected) graphs considered here, *L* is singular with a nullity of one. We note that graphical representations of materials microstructures have been employed by Johnson *et al.* to characterize grain-boundary networks^33^ and for microstructural design^34^.

**Figure 1:**
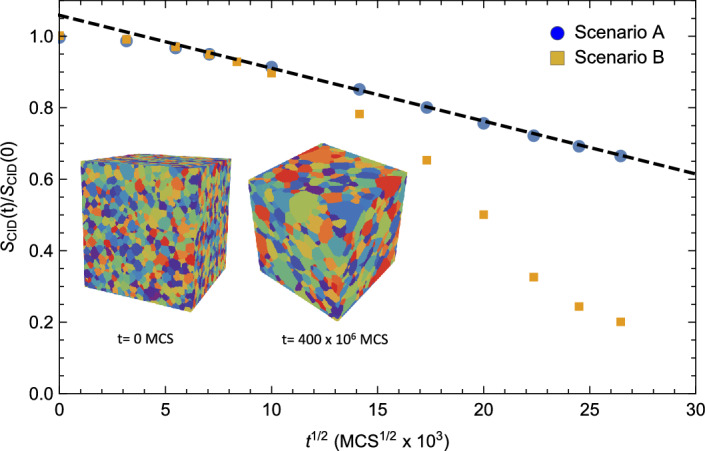
The computable information density $S_{CID}\left( t \right)$ relative to its initial value $S_{CID}\left( 0\right)$ as a function of $t^{1/2}$ for these two growth scenarios. The blue circles represent the information associated with Scenario A (normal, statistically self-similar grain growth^8^) while the gold squares represent the information associated with Scenario B (deviations from normal grain growth). Note that the CID for Scenario A scales as $t^{1/2}$ at late times, consistent with the statistically self-similar grain growth hypothesis, whereas the time dependence of the CID for Scenario B is more complex at late times. The inset shows snapshots of the microstructure for Scenario B at times $t = 0$ and $t = 400 \times 10^{6}$ MCS. (It should be noted that the initial microstructural data was created from a modified Voronoi construction and is therefore not representative of steady-state behavior).

We employ first the CID to analyze the temporal evolution of two evolving synthetic microstructures, as embodied in strings $x \left( t \right)$ and $y \left( t \right)$ that represent growth Scenarios A and B, respectively (see “Methods” section), that start from the same initial microstructure, denoted by $z \left( t \right)$, and coarsen as a function of time, *t*. Figure 1 illustrates the information density $S_{CID} \left( t \right)$ relative to its initial value $S_{CID} \left( 0 \right)$ as a function of $t^{1/2}$ for these two growth scenarios. As is evident from the figure, the CID for Scenario A varies as $t^{1/2}$ at late times, consistent with the statistically self-similar grain growth hypothesis, whereas the time dependence of the CID for Scenario B agrees with that of Scenario A at early times (as expected) and is more complex at late times. The deviation in $S_{CID} \left( t \right)$ values for these curves occurring at $t \approx 150 \times 10^{6}$ Monte Carlo steps (MCS) therefore indicates a concomitant deviation from statistical self-similarity, possibly due to AGG as it is conventionally defined. We will investigate this possibility in more detail below. Also shown in Fig. 1 are two microstructural snapshots, one at the beginning of the simulation and one after $t = 400 \times 10^{6}$ MCS for Scenario B. This second snapshot reveals AGG.

**Figure 2:**
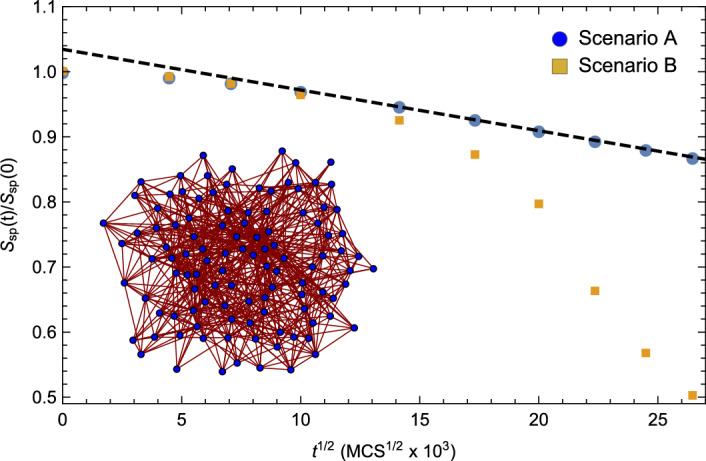
The relative spectral complexity, $S_{spec}\left( t \right) / S_{spec}\left( 0 \right)$ as a function of $t^{1/2}$. The blue circles represent the information associated with Scenario A (normal, statistically self-similar grain growth^8^) while the gold squares represent the information associated with Scenario B (deviations from normal grain growth). Again, note that the spectral complexity for Scenario A scales as $t^{1/2}$ at late times, consistent with the statistically self-similar grain growth hypothesis. The inset shows a graphical representation of the microstructural network at $t= 700 \times 10^{6}$ MCS for Scenario B. In this representation the vertices correspond to the distinct grains and the edges link the grains to their nearest neighbors.

For the purpose of comparison, the spectral complexity given in Eq. (1) was also calculated for the same time series of evolving microstructures (and in this case graph Laplacians) considered above. Figure 2 shows the resulting relative complexity, $S_{spec}\left( t \right) / S_{spec}\left( 0 \right)$ as a function of $t^{1/2}$ for the two aforementioned growth scenarios. The inset shows a graphical representation of the microstructural network at $t= 700 \times 10^{6}$ MCS for Scenario B. We note that, again, the spectral complexity for Scenario A scales as $t^{1/2}$ and that the complexity curves for the two scenarios begin to deviate at $t \approx 150 \times 10^{6}$ MCS. This time to deviation is consistent with that observed for the CID analysis above.

### Link with extreme events in the grain-size distribution

**Figure 3:**
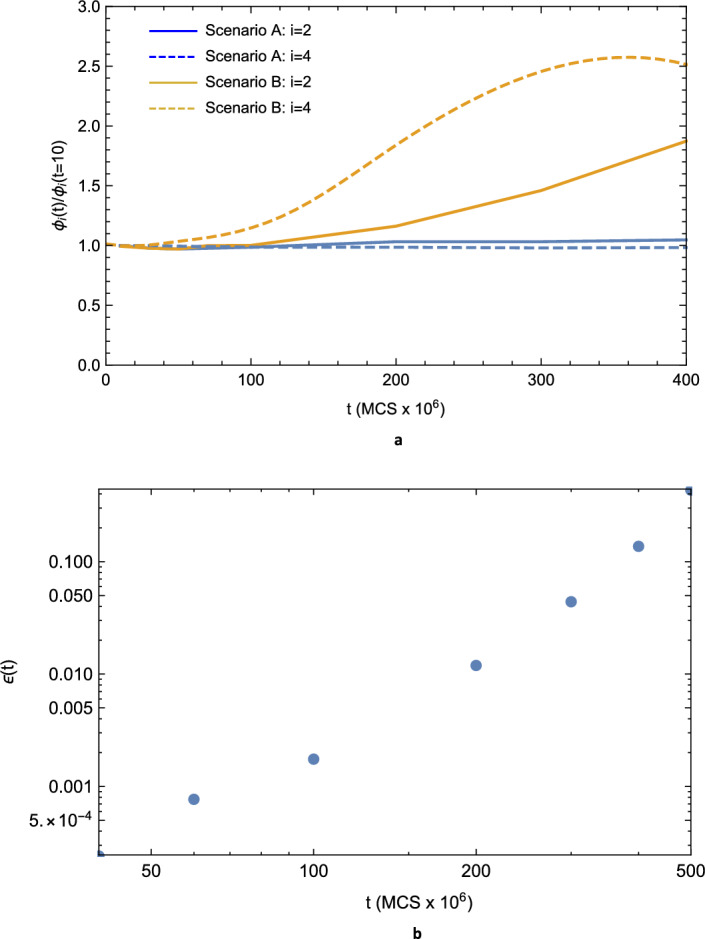
(**a**) The moments, $\phi _{i} \left( t\right) /\phi _{i} \left( t=10\right)$, relative to their values at $10 \times 10^{6}$ MCS of the grain-diameter pdf versus time, *t*, for Scenarios A (blue curves) and B (gold curves), respectively. The variance ($i=2$) and the kurtosis ($i=4$) are represented by the solid and the dashed curves, respectively. It should be noted that both blue curves are essentially time-invariant while the gold curves start to deviate from the corresponding blue curves at early times. (**b**) The exceedance, $\epsilon \left( t \right)$, as a function of time, *t*, for microstructures evolving according to Scenario B.

To determine more accurately the time scale for the onset of AGG and thereby which microstructural, and by extension substring and spectral, characteristics control the behavior of the entropy curves shown in Figs. 1 and 2 , we examine next the time dependence of two of the lower-order moments about the mean of the pdfs of the normalized, effective spherical grain diameters, $d \left( t \right) /{\bar{d}}\left( t \right)$, where ${\bar{d}} \left( t \right)$ is the time-dependent mean of the grain diameter, $d \left( t \right)$. For the case of statistically self-similar growth, the normalized *i*-th moments, $\phi _{i} \left( t\right)$, of a given pdf should be time-invariant. Figure 3a displays the normalized variance ($i=2$) and kurtosis ($i=4$) for Scenarios A and B as a function of time, the former quantifying the (square of the) dispersion of the pdfs and the latter characterizing their tailedness (i.e., frequency of outliers). It should be noted that both curves for Scenario A are essentially time-invariant, as expected, while the curves for Scenario B start to deviate from their counterparts at early times. Since $\phi _{4} \left( t=10\right) \approx 2.8$ for both scenarios, the monotonic increase in $\phi _{4} \left( t\right)$ with time for Scenario B indicates that the corresponding distribution is becoming fat-tailed, or leptokurtic (i.e., having significant outliers) over time^35^. As the onset of the deviation in $\phi _{4} \left( t\right)$ occurs at approximately $50 \times 10^{6} - 60 \times 10^{6}$ MCS and somewhat before the corresponding onset in the deviation in $\phi _{2} \left( t\right)$, it is sensible to identify this range of times with the onset of AGG.

To quantify further abnormality in the microstructural grain-size distribution, one regards abnormal grains as rare events that may be described by the properties of the tail of the distribution. Then, using the formalism of extreme-event statistics employed to analyze risk in financial markets, one can define analogous tools here, including conditional tail moments and the exceedance^9,36,37^. The exceedance, $\epsilon$, is particularly useful. For a probability density function of grain volume, *V*, given by $p\left( V \right)$,

2 $$\begin{aligned} \epsilon {:}{=}\int _{V_{c}}^{\infty } dV \; p\left( V \right) , \end{aligned}$$

where $V_{c}$ is a critical grain volume that marks the start of the tail of the distribution and is conventionally chosen as ten times the average grain volume at $t=0$^38^. We determined $p\left( V \right)$ from grain volume histograms compiled from the Potts model simulations. Figure 3b shows the behavior of $\epsilon$ as a function of *t* for the microstructure evolving under Scenario B. It should be noted that at $t = 100 \times 10^{6}$ MCS $\epsilon \approx 0.002$ with several grains having $V > 20 V_{c}$.

### Metrics and shared information for evolving microstructures

Several quantities based on the compressor ${\mathcal {C}}$ and the spectrum of *L* facilitate microstructural interrogation and comparison. We focus first on quantities that exploit the properties of the compressor (see the “Methods” section below). For example, the normalized compression distance (NCD) between two strings *x* and *y*, each representing a particular microstructure, measures the difference between the two files and is given by

3 $$\begin{aligned} \text {NCD} \left( x,y\right) {:}{=}\frac{{\mathcal {C}} \left( x y\right) - \text {min} \{ {\mathcal {C}} \left( x \right) ,{\mathcal {C}} \left( y\right) \} }{\text {max} \{ {\mathcal {C}} \left( x \right) ,{\mathcal {C}}\left( y\right) \}}, \end{aligned}$$

where max(min) denotes the maximum (minimum) of a list. We note that NCD is a distance metric^39^ such that $0 \le \text {NCD} \left( x,y\right) \le 1+ e$, where smaller values indicate greater similarity between microstructures, and that *e* is a small number resulting from imperfections in the compressor^40^.

Similarly, one can also define the mutual information^41,42^ shared by two microstructures *x* and *y* in terms of the compressor ${\mathcal {C}}$ as

4 $$\begin{aligned} I \left( x,y\right) {:}{=}{\mathcal {C}} \left( x \right) +{\mathcal {C}} \left( y \right) -{\mathcal {C}} \left( xy\right) . \end{aligned}$$

We will also wish to assess the information contained in *x* and *y* in cases where there may be a causal chain linking these microstructures to a third (starting) microstructure, characterized by a string *z*. It is of interest to determine whether the link between *x* and *y* is direct or is influenced by the mutual link with *z*. For this purpose, it is useful to introduce the partial mutual information^42^

5 $$\begin{aligned} I^{\prime } \left( x,y,z\right) {:}{=}{\mathcal {C}} \left( xz\right) +{\mathcal {C}} \left( yz\right) -{\mathcal {C}} \left( z\right) -{\mathcal {C}} \left( xyz\right) , \end{aligned}$$

which represents the information shared between *x* and *y* that is not contained in *z*, and the associated interaction information^43,44^

6 $$\begin{aligned} {\mathcal {I}} \left( x,y,z\right) = I \left( x,y\right) -I^{\prime } \left( x,y,z\right) . \end{aligned}$$

**Figure 4:**
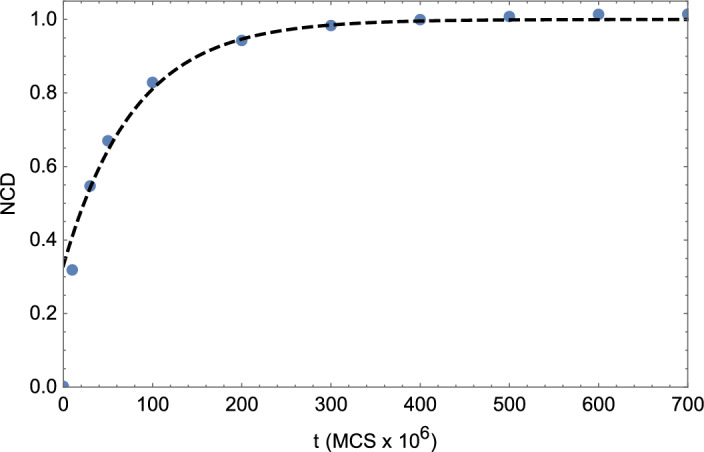
The normalized compression distance (NCD) for normal grain growth (i.e., Scenario A) relative to the starting microstructure versus time, *t* (blue circles). The dashed line represents the best exponential fit to the data (apart from the initial state and as shown in the figure).

The interaction information may be either positive or negative and reflects, in this context, the information shared between microstructures *x* and *y* after eliminating the contributed information that is conditioned on both originating from a given initial microstructure *z*. In this context, a positive interaction indicates that the correlation between microstructures evolving under the two different scenarios (i.e., A and B) depends in part on the fact that they both started from the same initial microstructure. Given the decrease in microstructural information that attends grain growth that is highlighted in Fig. 1, it is useful to obtain an effective information correlation time, $\tau$, associated with coarsening. For this purpose, we next consider the time evolution of the NCD for normal grain growth (i.e., Scenario A) relative to the starting microstructure, as illustrated in Fig. 4. By obtaining the best exponential fit to the data as given by $NCD \left( t \right) = \exp {\left( -t/\tau _{NCD} \right) }$, one finds that $\tau _{NCD} = 79.2 \times 10^{6}$ MCS. In this context $\tau _{NCD}$ represents the characteristic time over which information from the initial state propagates during grain growth. We note that the analog of the NCD for the microstructural graphs discussed above is the spectral distance between graphs^45^. This quantity is readily calculated from the differences in graph eigenvalues, but won’t be considered here.

**Figure 5:**
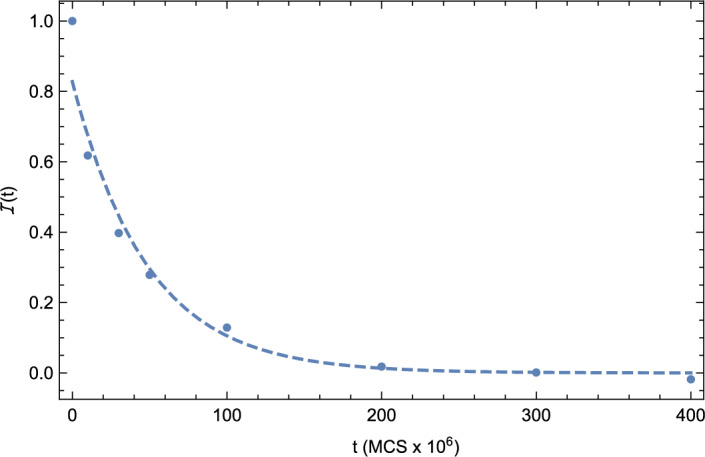
The interaction information, ${\mathcal {I}}$, as a function of time, *t*, for the growth scenarios considered here. The dashed line represents the best exponential fit to the data (apart from the initial state and as shown in the figure).

It is now possible to assess the degree to which mutual information is shared between microstructures evolving under the different scenarios described here. As both evolutionary paths begin with the same starting microstructure, it is informative to obtain the partial mutual information, or the corresponding interaction information, that eliminates the shared information resulting from the common starting point. Figure 5 shows the interaction information, ${\mathcal {I}} \left( x,y,z\right)$, as a function of time for the growth scenarios considered here. A characteristic correlation time $\tau _{{\mathcal {I}}}$ for the two scenarios can be obtained from an exponential fit to the data, with the result that $\tau _{{\mathcal {I}}}=48.5 \times 10^{6}$ MCS. One interpretation of $\tau _{{\mathcal {I}}}$ is that it is a characteristic time associated with the deviation from normal grain growth. This interpretation is in agreement with the estimated onset time range for AGG based on the time-dependent behavior of the kurtosis, as described above.

## Discussion

We examined the time-dependent information content of synthetic coarsening microstructures using both a computable information density (CID) based on file compression and a spectral entropy along with selected metrics and measures of shared information and interaction strength. These approaches permit one to identify in a *dynamic* context deviations from normal grain growth and to obtain correlation times associated with different coarsening scenarios, including those that exhibit abnormal grain growth. In particular, we identified a characteristic time associated with the deviation from normal grain growth that is associated with complexion-mediated growth and highlighted a temporal regime associated with abnormality. The methods employed here are, of course, not restricted to any particular growth mechanism and may be used to interrogate dynamically systems having inhomogeneous grain-boundary energies and/or mobilities. We emphasize that this work provides a prototypical example of the application of complexity (entropy) production to characterize a ubiquitous non-equilibrium phenomenon, namely grain growth. Moreover, the approach employed here preserves to a large degree important microstructural characteristics, including voxel neighbor information, and is therefore a faithful representation of overall information content. One possible extension of this work is the use of the calculated entropy production in an irreversible thermodynamics framework to identify, for example, forces and fluxes that govern the evolution of a system.

Given the generality of the methodology described here, we expect that this approach may be used to distinguish among various scenarios associated with abnormal grain growth. For example, beyond complexion-mediated growth, other mechanisms, such as grain-boundary energy inhomogeneity and boundary pinning, may lead to abnormal growth regimes with different temporal dependencies and therefore different evolving information complexities. Both the CID and the spectral entropy embody these differences. We also expect that the graph Laplacian spectrum used to compute the spectral entropy may contain additional useful information reflecting spatio-temporal correlations among growing abnormal grains. The use of microstructural complexity to explore the impact of abnormal grain growth mechanisms is the subject of ongoing work.

### Experimental data for thin-film growth

**Figure 6:**
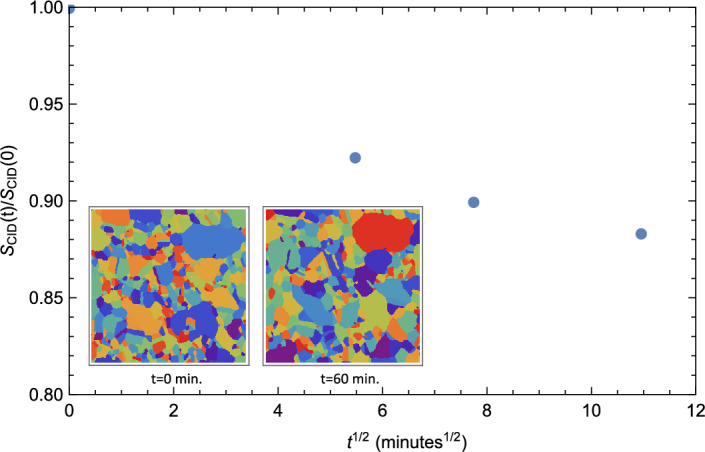
The computable information density $S_{CID}$ relative to its initial value $S_{CID}\left( 0\right)$ as a function of $t^{1/2}$ for a coarsening polycrystalline Pd thin film held at a temperature of $T = 400^{0}$C. The inset shows the corresponding microstructures at $t=0$ and 60 minutes. The field of view has dimensions of 1196 nm $\times$ 1196 nm.

To illustrate the utility of this approach, we consider next the time evolution of a coarsening experimental microstructure, namely a thin Pd film held at $T = 400^{0}$C for 120 minutes. As the film comprises nearly columnar grains, we examine a series of 2d microstructures and focus on the time evolution of the CID. Figure 6 displays the computable information density $S_{CID}\left( t \right)$ relative to its initial value $S_{CID}\left( 0\right)$ as a function of $t^{1/2}$ for this film. As was the case for the synthetic microstructures considered above, $S_{CID}\left( t \right)$ decreases monotonically with *t*, though the limited amount of data precludes making any conclusions regarding the existence of a normal regime. However, even with this limited data, one can deduce that the characteristic time over which information from the initial state propagates during grain growth, $\tau _{NCD}< 30$ minutes since $NCD(30 \; \textrm{min}) \approx 0.98$. Thus, while Fig. 6 indicates that a moderate amount of coarsening occurred over the course of the experiment, concomitant changes in grain shape, etc., lead to relatively small correlation times.

### Complexity, entropy and thermodynamic analogies

**Figure 7:**
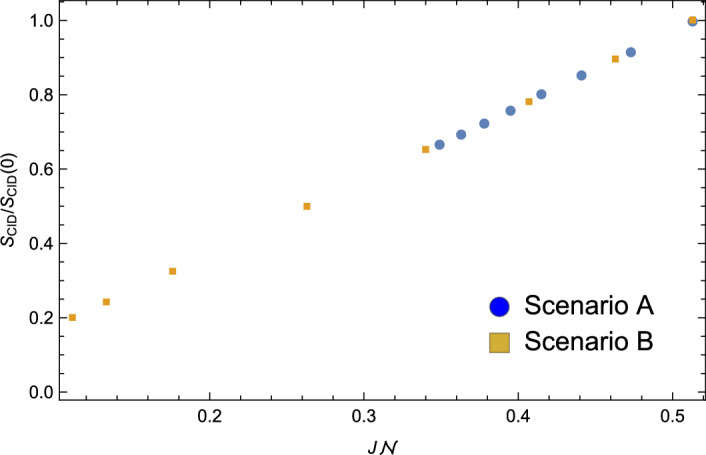
The computable information density $S_{CID}$ relative to its initial value $S_{CID}\left( 0\right)$ versus the internal energy $U = J {\mathcal {N}}$ ($J=1$) for Scenarios A(blue circles) and B (gold squares).

Given the relationship between complexity and entropy, it is useful to explore related thermodynamic analogies associated with coarsening^20,24^. By analogy with the thermodynamic analysis of time-evolving networks by Ye *et al.*^24^, one can define the internal energy, *U*, of a microstructure as proportional to the total grain-boundary area or, in the language of graphs, proportional to the number of edges, ${\mathcal {N}}$. This definition is fully consistent with the internal energy, $U = J {\mathcal {N}}$ obtained in a Potts model with constant energy parameter *J* (see “Methods” section). With this definition, one can then also define an (inverse) temperature, $1/T = \partial S/\partial U$, associated with the microstructure^46^. We focus again on the CID. Figure 7 shows the computable information density ratio $S_{CID}/S_{CID}\left( 0 \right)$ versus the internal energy $U = J {\mathcal {N}}$ ($J=1$) for Scenarios A and B. As is evident from the figure, $S_{CID}$ depends linearly on *U*, resulting in a constant positive microstructure temperature *T*. This is perhaps not surprising since the presence of a grain-boundary segment corresponds to a break in a substring, and so while the time-dependence of $S_{CID}\left( t\right)$ depends on the growth scenario, $S_{CID} \left( U \right)$ depends only on *U* for either scenario.

Finally, we note that there are alternative descriptions of complexity in which a microstructure is described in terms of correlations of an underlying point process. For example, in a recent paper^47^, we quantify the entropy of a microstructure in terms of a two-point correlation function (the radial distribution function) of grain triple junctions. From the radial distribution function one can extract a so-called direct correlation function that may be employed in a classical density-functional model of microstructure evolution.

## Methods

### File compression and algebraic complexity

As has been well established, one can apply a compression algorithm to a string *x*, such as that represented by ${\mathcal {C}} \left( x\right)$, as an approximation to the Kolmogorov complexity, $\text {K} \left( x\right)$. We employ for ${\mathcal {C}}$ the Unix-based xz lossless compressor that exploits the Lempel-Ziv-Markov chain algorithm (LZMA). This utility is based on LZ77 (the first simple compression algorithm due to Ziv and Lempel^48^) and employs a sliding dictionary algorithm and a filter to render the data suitable for compression with the dictionary^49^. In particular, it was found that xz -8 had a dictionary size that was satisfactory for the applications here. ${\mathcal {C}}$ is a so-called normal compressor as it possesses the following important properties (up to an additive term) that permit one to define meaningful metrics, etc.: ${\mathcal {C}} \left( xx\right) ={\mathcal {C}} \left( x\right)$ (idempotency), ${\mathcal {C}} \left( xy\right) \ge 0$ (monotonicity), ${\mathcal {C}} \left( xy\right) ={\mathcal {C}} \left( yx\right)$ (symmetry), ${\mathcal {C}} \left( xy\right) +{\mathcal {C}} \left( z\right) \le {\mathcal {C}}\left( xz\right) +{\mathcal {C}} \left( yz\right)$ (distributivity)^50^. It should be noted that the string product *xy* implies string concatenation.

### Monte Carlo simulations of grain growth

Monte Carlo simulations of three-dimensional coarse-grained microstructures on a voxelated lattice were performed using a modified *Q*-state Potts model in which interfacial phase (complexion) transitions^12^ may occur as both correlated and uncorrelated stochastic events that modify grain-boundary mobilities and thereby evolving microstructures over some period of time^25,38^. Each voxel represents a coarse-grained piece of the system comprising a very large number of particles, and neighboring voxels that share an spin value $S \left( 1 \le S \le Q \right)$ constitute a single grain. The Hamiltonian for this system is given by

7 $$\begin{aligned} H = -J \sum _{\langle i,j \rangle } \left( \delta _{S_{i},S_{j}} -1\right) , \end{aligned}$$

where where *i* and *j* refer to voxels, $J>0$ is a (constant) energy parameter, the angle brackets denote distinct nearest-neighbor voxel pairs and $\delta$ is the Kronecker delta. Thus, neighboring unlike spins are associated with an energy cost, and so the time evolution of the model based on a modified Metropolis rule^25,51^ at fixed, artificial inverse temperature $\beta$ leads to an increase in average grain diameter. As is customary, time is measured in Monte Carlo steps (MCS).

As summarized above, for the grain growth simulations, two prototypical grain growth scenarios following from different complexion nucleation assumptions are modeled here, namely: (a) no complexion transitions, leading to isotropic, normal grain growth (NGG) that is statistically self-similar^8^ and (b) complexions are spatially randomly nucleated via a cooperative mechanism. More specifically, in these simulations, complexions are randomly nucleated on grain boundaries and are propagated to nearby grain boundaries if neighboring grain boundaries have already transitioned (case b). This is the double-adjacency mechanism is described by Frazier et al. and Marvel et al.^25,38^. The nucleation rate was 2 complexion transitions for every 50, 000 MCS, corresponding to a temperature of 1450 $^{\circ }$C and an activation energy of 384 kJ per mole. This temperature was chosen to match a reference temperature in an experimentally-obtained Eu-doped MgAl$_{2}$O$_{4}$ complexion time-temperature-transformation (TTT) diagram^16^.

We note that the hypothesis of statistical self-similarity implies that the probability density function (pdf) of the grain diameter of evolving configurations may be recast into a time-independent, scale-invariant form. In case (b), complexion nucleation alters grain-boundary mobilities and results in deviations from NGG. Abnormal grain growth (AGG) sometimes occurs during coarsening after an initial incubation period and we are particularly interested in this outcome here.

### Sputtered Pd thin film

With regard to the experimental microstructures highlighted in the Discussion section, the Pd films were deposited via DC magnetron sputtering at a power of 50W and at a pressure of 1.1 mTorr of argon, yielding a nominal sputtering rate of 0.051 nm/s and films with a nominal thickness of 30 nm. The substrate was a MEMS heating chip with an electron transparent silicon nitride window that was held at room temperature during deposition. The film was pre-annealed to achieve a columnar microstructure. The coarsening experiment was performed *in situ* in the transmission electron microscope at $T = 400^{\circ}$C for a total of 120 minutes. The sample was periodically quenched to room temperature and the microstructure was recorded via precession enhanced electron diffraction based orientation mapping. Using the TSL OIM (©) software package, “Grain Dilation” and “Single Orientation per Grain” cleanup operations were applied. For each time step, grains were identified from the cleaned orientation maps with a tolerance angle of $5^{\circ}$.

## Data Availability

The authors will make available, upon request, the data used in this work. It is understood that the data provided will not be for commercial use. Those interested in acquiring the data should contact J. M. Rickman (jmr6@lehigh.edu).

## Code availability

The authors will make available, upon request, the code used in the applications described in this work. It is understood that the code will not be for commercial use.
